# A Comparative Investigation of Machine Learning Algorithms for Pore-Influenced Fatigue Life Prediction of Additively Manufactured Inconel 718 Based on a Small Dataset

**DOI:** 10.3390/ma16196606

**Published:** 2023-10-09

**Authors:** Bing-Li Hu, Yan-Wen Luo, Bin Zhang, Guang-Ping Zhang

**Affiliations:** 1 Shenyang National Laboratory for Materials Science, Institute of Metal Research, Chinese Academy of Sciences, 72 Wenhua Road, Shenyang 110016, China; blhu21s@imr.ac.cn; 2 School of Materials Science and Engineering, University of Science and Technology of China, Shenyang 110016, China; 3 Key Laboratory for Anisotropy and Texture of Materials, Ministry of Education, School of Materials Science and Engineering, Northeastern University, 3-11 Wenhua Road, Shenyang 110819, China

**Keywords:** small dataset, machine learning, algorithm, fatigue life, additive manufacturing

## Abstract

Fatigue life prediction of Inconel 718 fabricated by laser powder bed fusion was investigated using a miniature specimen tests method and machine learning algorithms. A small dataset-based machine learning framework integrating thirteen kinds of algorithms was constructed to predict the pore-influenced fatigue life. The method of selecting random seeds was employed to evaluate the performance of the algorithms, and then the ranking of various machine learning algorithms for predicting pore-influenced fatigue life on small datasets was obtained by verifying the prediction model twenty or thirty times. The results showed that among the thirteen popular machine learning algorithms investigated, the adaptive boosting algorithm from the boosting category exhibited the best fitting accuracy for fatigue life prediction of the additively manufactured Inconel 718 using the small dataset, followed by the decision tree algorithm in the nonlinear category. The investigation also found that DT, RF, GBDT, and XGBOOST algorithms could effectively predict the fatigue life of the additively manufactured Inconel 718 within the range of 1 × 10^5^ cycles on a small dataset compared to others. These results not only demonstrate the capability of using small dataset-based machine learning techniques to predict fatigue life but also may guide the selection of algorithms that minimize performance evaluation costs when predicting fatigue life.

## 1. Introduction

Aircraft turbines, compressors, turbochargers, and further components for which high-temperature strength and creep resistance are required have been widely used [1]. However, the shaft connecting the turbine disc must have adequate fatigue strength. The demand can be fulfilled by obtaining joints with dissimilar metals [2]. The nickel-based alloy Inconel 718 is a promising and widely investigated material for additive manufacturing (AM), which is mostly processed by laser powder bed fusion (LPBF). This alloy offers good weldability, good fatigue, and creep performance values at elevated temperatures, which is of real interest to researchers [2,3].

LPBF has been recognized as a promising AM technique due to its flexibility in feedstock and shapes [4]. Although great progress in LPBF processing has been made, the poor fatigue performance and pronounced lifetime scatter of fabricated metallic components due to manufacturing process-induced inherent defects impede the widespread and long-term adoption of components [5,6]. Representative manufacturing defects mainly include surface roughness, gas pores, and lack-of-fusion (LOF) porosity [5,7]. These defects, as uncertain influencing factors, have a detrimental role in fatigue performance and lead to a large scattering and uncertainty of the fatigue life [8]. The quantitative relationship between fatigue life and features of the manufacturing defect population has attracted increasing attention in both academic and industrial communities [9]. The location, shape, and size of internal defects are the primary variables for the decrease in the HCF life of AM metallic materials, and the location is considered to have a more adverse effect as opposed to the shape and size because crack initiation sites are mostly observed close to the specimen surface [10]. The severity of the effect can be ranked in terms of the decreasing significance of the location size, shape, or orientation [11,12]. Yadollahi et al. [13] reported that fatigue cracks originated from the pores of the parts, and fatigue life was directly affected by the pore features of the AM parts, such as pore size and shape. Hu et al. [9] conducted a detailed investigation into the influence of manufacturing porosity and lack-of-fusion defects on the fatigue resistance of selective laser melting-processed Ti-6Al-4V. It has also been proposed that the combined effects of the location, size, and shape determine the most dangerous defect [14]. The sample-to-sample variation in fatigue life has also been shown to be related to the geometric aspects of these critical defects [6].

To further clarify the quantitative relationship between defects and fatigue life, numerical simulations, analytical solutions, and experimental measurement methods have been adopted by numerous researchers. Bergara et al. [15] presented the numerical simulation and validation of a fatigue propagation test of a semielliptical crack located at the side of the rectangular section of a beam subjected to four-point bending. Shen et al. [16] proposed a prediction method based on a virtual simulation, employing the nominal stress method and Miner’s cumulative damage theory to calculate the gear contact fatigue life based on a modified material P-S-N curve. Nevertheless, more scientific, efficient, and accurate methods for predicting fatigue life are urgently needed.

Machine learning (ML) techniques can save a considerable amount of time in scientific experiments and technical development, greatly improving research efficiency and the quality of newly developed material structures. ML is an innovative data-driven approach that has been increasingly applied to predict the fatigue life of components, with a growing number of studies reporting successful outcomes [17,18,19]. Feng et al. [17] applied ML approaches to check the important factors involved in predicting fatigue strength, providing constructive guidance for the future development of fatigue-resistant steel materials. Luo et al. [20] clarified the quantitative correlation between the fatigue life of AM-fabricated components and their pore features, such as the location, size, and number of pores, through ML approaches. Bao et al. [17] showed that the location, size, and shape of defects in Ti-6Al-4V samples also affected the fatigue life of components to varying degrees. Additionally, Zhou et al. [21] reported that five genetic features were identified by optimizing the fatigue life prediction model for 316LN specimens under different uniaxial and multiaxial loading paths. As ML techniques continue to evolve, they not only enable the prediction of the quantitative relationship between fatigue life and features but also facilitate the identification of key features that affect fatigue life.

To develop accurate ML prediction models, it is essential to build them based on a sufficient dataset. Generally, obtaining a large quantity of experimental data through conventional fatigue tests, particularly in HCF experiments, can be prohibitively expensive and may not be feasible for several research projects. The predictive performance of different ML algorithms can vary depending on the size of the dataset, with models trained on smaller datasets generally exhibiting lower accuracy than those trained on larger and more diverse datasets [22]. Feng et al. [23,24] showed that deep neural network algorithms trained on small datasets typically performed worse than some ML algorithms such as shallow neural networks and support vector machines. The challenge of collecting and assembling large datasets is a limiting factor that hampers the widespread adoption of ML techniques in materials science [24]. Expanding the use of ML techniques for fatigue life prediction requires the development of methods that can handle small datasets effectively. In cases where additional data cannot be obtained, it is crucial to fully leverage the available small dataset and select appropriate ML algorithms to build more accurate models. This underscores the importance of careful algorithm selection and feature engineering in the absence of large datasets.

In recent years, the field of ML has seen a growing interest in the study of algorithms [25]. Previous research has proven that the selection of ML algorithms significantly impacts both the accuracy and generalization ability of the prediction model [26]. For instance, Luo et al. [20] applied three basic ML algorithms to a small dataset to establish a quantitative relationship between pore features and fatigue life. Unfortunately, the predictive performance of the three ML algorithms (linear regression, support vector regression, and kernel ridge regression) was observed to be suboptimal in a previous study, and the coefficient of determination (*R*^2^) was between 0.6 and 0.7. Moreover, the generalizability of other ML algorithms on the small dataset of pore-affected fatigue life remains unclear and requires further investigation. Future research can focus on correcting deviations in prediction models by choosing a reasonable algorithm. Additionally, it would be beneficial to investigate the effectiveness of various algorithms for small datasets, as this greatly enhances the predictive capability of these models and advance the field even further. While these datasets [20,27] are smaller than what is typically used in materials research, it is believed that small datasets still hold potential for application in fatigue life prediction. Zhang et al. [23] proposed a strategy for applying ML algorithms to small datasets in materials science. The investigation was conducted to determine whether other ML algorithms could enhance the accuracy of predicting pore-affected fatigue life to accurately explore the effect of pores on fatigue life.

In this work, the small dataset of pore-affected fatigue life of LPBF-fabricated Inconel 718 reported by Luo et al. [20] was trained and modeled for the prediction of fatigue life employing thirteen ML algorithms, namely adaptive boosting (ADABOOST), artificial neural network (ANN), decision tree (DT), extra tree (ET), elastic net regression (ENR), gradient boosting decision tree (GBDT), least absolute selection and shrinkage operator regression (LASSO), linear kernel function-support vector regression (L-SVR), multiple linear regression (MLR), polynomial regression (PR), ridge regression (RIDGE), random forests (RF), and extreme gradient boosting (XGBOOST). After training these models, they were evaluated using test sets. The evaluation results of the prediction models established by the thirteen ML algorithms were repeatedly verified on virtual test sets generated through random partitioning using the high probability principle, and the ranking results of the algorithms were consequently obtained.

## 2. Experimental Methods

### 2.1. Specimen Preparation and Fatigue Testing

**Figure 1:**
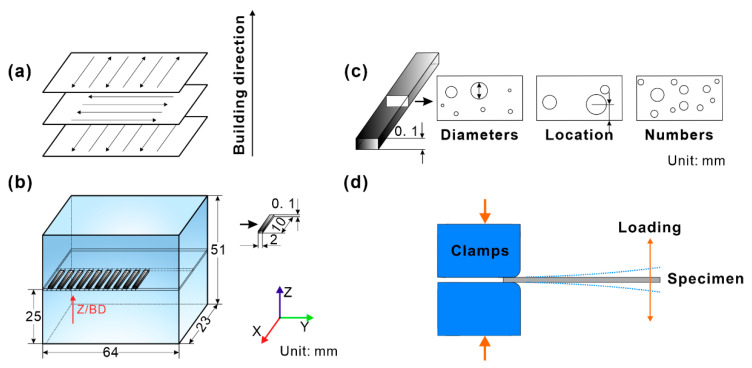
Schematic illustrations of (**a**) the scanning strategy with a 90° rotation between layers; (**b**) orientations, grouping, and dimensions of specimens extracted from LPBF-fabricated Inconel 718 block; (**c**) different pore features, including diameter, location, and number of pores; (**d**) symmetrical bending fatigue of cantilever beam specimen.

An Inconel 718 prismatic component with dimensions 64 × 23 × 51 mm^3^ was fabricated on an EOSINT M280 machine equipped with a 400 W laser fiber utilizing the LPBF method. The detailed fabrication process for the Inconel 718 prismatic component has been reported previously [20]. To obtain a block with a high density and a low residual stress [28,29], a scanning strategy with a rotation angle of 90° between each layer was adopted [30,31], as schematically illustrated in Figure 1a. To examine the potential difference in fatigue life induced by pores at the different locations of the Inconel 718 prismatic component, we adopted small-scale cantilever beam specimens taken from different locations in the as-built Inconel 718 prismatic component [20]. All the beam specimens were cut from the same height of 25 mm in different places using a spark-cutting machine, as shown in Figure 1b. AM parts in the as-built state always possess a significantly large surface roughness and many defects due to the layer-upon-layer build strategy during the AM processing. These factors may degrade the fatigue properties, especially the HCF properties [32]. After machining and polishing treatments, the fatigue properties of LPBF 316L increased by 50% compared with those of as-built ones, and similar results were also reported for other AM metallic materials [32]. Then, these cantilever beam specimens were ground mechanically and electropolished to obtain smooth surfaces and eliminate the surface residual stress induced by the spark-cutting process.

The build thickness effect on the fatigue properties of AM parts still needs to be elucidated. These influencing factors not only include the intrinsic factors related to microstructures but also the extrinsic factors associated with defects [32]. This study mainly discusses the influence of pore features on fatigue life. To avoid the size effect caused by thickness on fatigue life, it was necessary to frequently measure the sample size during mechanical processing and polishing to ensure that the actual thickness of the final fatigue sample was within the standard specification thickness error range. The final dimensions of the cantilever beam specimens were 10 × 2 × 0.1 mm^3^ [20]. Figure 1c depicts the pore features (diameter, location, and numbers) in the cantilever beam specimen.

After controlling the variables, preparations were made for the next step of fatigue testing dominated by pores. Fatigue tests were carried out at room temperature using a self-developed, patented, small cantilever beam machine [20,33], which has been proven to be suitable for evaluating the fatigue properties of various cantilever beam specimens [27,34,35,36]. The cantilever beam specimens were mainly subjected to symmetric bending fatigue loading under constant deflections of approximately ±0.8 mm and ±0.85 mm, respectively, corresponding to fixed strain amplitudes of about 0.274 and 0.295 [20,33,37]. The strain ratio and sinusoidal wave loading frequency were set as −1 and 50 Hz, respectively. More detailed information on symmetric bending fatigue testing of the cantilever beam specimens can be found in our previous work [20,33,37]. Figure 1d schematically illustrates the fatigue loading of the cantilever beam specimen.

### 2.2. Finite Element Simulation Method

The stress distribution of the cantilever beam specimens under a constant deflection of 0.65 mm was analyzed by the finite element method. The model of the entity shown in Figure 1d consisted of clamps and a specimen. A set of clamps were set as rigid bodies, and Young’s modulus and Poisson’s ratio of the specimen were defined as 180 GPa and 0.3, respectively. The friction parameter of the contact surfaces between the specimen and the two clamps was set as 0.1. The upper clamp was subjected to a constant load of 500 N to avoid the sliding of the specimen during the deformation. These conditions were adopted for the finite element analysis (FEA).

### 2.3. Microstructure Characterization

Microstructures of the specimens were characterized by an optical microscope (OM), an electron backscatter diffraction (EBSD) detector mounted on a field-emission scanning electron microscope (FE-SEM, LEO Supra 35, Oberkochen, Germany), and a transmission electron microscope (TEM, FEI Tecnai20, Houston, TX, USA) [20]. The void distribution of the specimens was also examined by three-dimensional X-ray tomography (3D-XRT, Xradia Versa XRM-500 system, Pleasanton, CA, USA). The SEM characterization of pore features in fatigued specimens and a simulation result of stress distribution in the specimens with different pore features indicated that the shape of almost all pores was spherical [20]. Therefore, the pores of the specimens were characterized by sphericity, as shown in Figure 1c.

### 2.4. Dataset Acquisition

By using the above cantilever beam specimen tests and microstructure characterization, the effective fatigue test dataset was obtained. Table 1 presents an overview of the dataset ranges for twenty-eight as-built specimens. To investigate the feasibility of popular ML algorithms for fatigue life prediction using such a small dataset, a set of information on defects in the specimens made from LPBF-fabricated Inconel 718 was collected. Here, fatigue data obtained by the fatigue testing mentioned above using twenty-eight cantilever beam specimens [20,27] were selected for building the ML models, thus the twenty-eight specimens may contain different types of pore features. In the small dataset, five variables of the dataset were selected as defect features for learning, and more relevant information is presented in Table 1.

## 3. Machine Learning Methods

### 3.1. Machine Learning Framework

**Figure 2:**
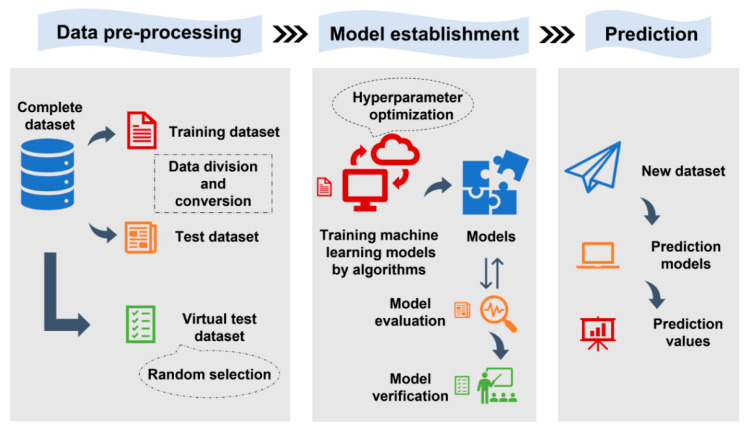
Strategy flowchart for ML techniques that use a small dataset to predict the pore-affected fatigue life of the AM-fabricated materials.

Figure 2 presents a strategy flowchart for predicting the pore-affected fatigue life of the AM-fabricated materials using a small dataset-based ML approach. The proposed framework has potential applications in the quality control and material selection of AM processes. By incorporating microscale features of defects, the method offers insight into the influence of pores on the fatigue performance of materials, which is critical for AM part design and optimization.

### 3.2. Data Preprocessing

The influence of pores far from the surface on fatigue performance is relatively small [20]. To better characterize the location information of pores, infinity (1 × 10^10^) was taken as the location value with zero pores. The statistical values of pore location in Table 1 contained a large order of magnitude and were normalized and standardized later. After checking and filtering the dataset, twenty-six preprocessed data points were obtained to build ML models following the steps below.

**Figure 3:**
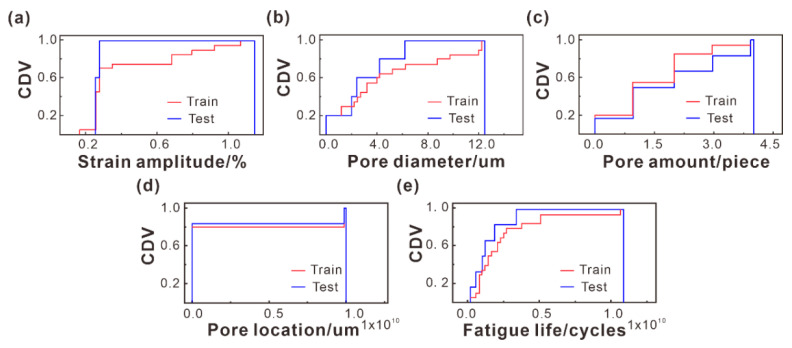
CDF diagrams of (**a**) strain amplitude (*ε*), (**b**) pore diameter (*d*), (**c**) pore amount (*m*), (**d**) pore location (*l*), and (**e**) fatigue life (*N*). The Y-axis represents the cumulative distribution values (CDV), and the X-axis represents the different pore features.

**Figure 4:**
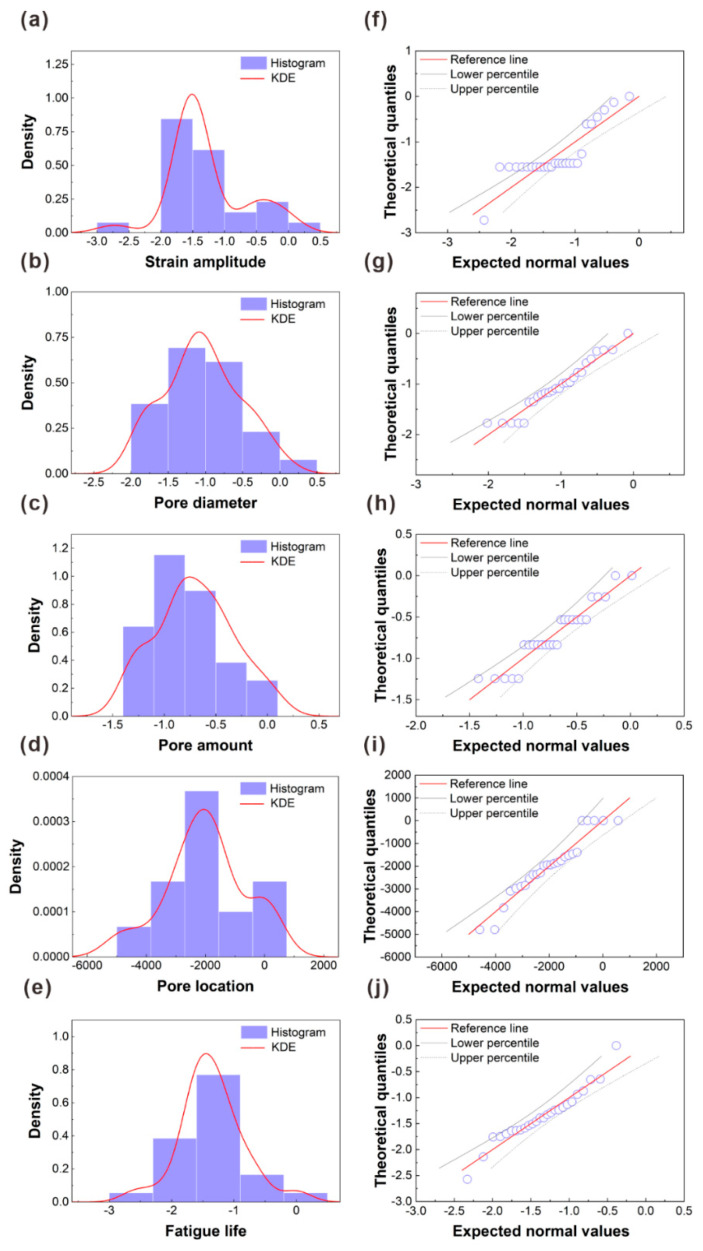
Gaussian distribution histograms and kernel density curves of the (**a**) strain amplitude (%), (**b**) pore diameter (µm), (**c**) pore amount (piece), (**d**) pore location (µm), and (**e**) fatigue life (cycles). The Q-Q plots of the (**f**) strain amplitude (%), (**g**) pore diameter (µm), (**h**) pore amount (piece), (**i**) pore location (µm), and (**j**) fatigue life (cycles), the circle points indicate the sample data.

1. Division of dataset: First, 80% of the samples were selected randomly as a training dataset and 20% as a testing dataset. The Kolmogorov–Smirnov (K–S) test was then adopted to use the cumulative distribution function (CDF) for comparison of the consistency of distributions between the training and test sets under different features, as shown in Figure 3. The results indicated that the distribution of the training and test sets was approximately consistent. For more explanatory information, see Supplementary comments to Figure 3.

2. Data normalization: There are many ways to normalize data, and here, a simple max-min normalization method is adopted to standardize the fatigue dataset. More information on the simple max-min normalization method is present in Supplementary Information (SI) and shown in Figure S1.
3. Normal transformation: Figure S2 shows the Gaussian distribution for each feature in the normalized dataset. The results indicate that some features exhibit significant skewness issues, and detailed explanations and correction methods are shown in Figure S2 of SI. Figure 4 displays the Gaussian distribution of features in the dataset after normalization and Box–Cox transformation [38]. From Figure 4a–e, it is evident that the skewness of the features is improved compared to the distribution in Figure S2a–e in SI. Furthermore, compared to Figure S2f–j in SI, the data points for each feature more closely approximate the theoretical normal distribution line, as shown in Figure 4f–j, indicating the normality of the dataset was enhanced after the Box–Cox transformation.

### 3.3. ML Techniques

To find the best ML algorithm for fatigue problems from the different ML algorithms, Table 2 summarizes the details of various ML algorithms for fatigue life prediction on the small dataset, as adopted here.

Some typical individual algorithms, bagging [58] and boosting [59] algorithms for ensemble learning [60] are introduced and divided into four categories to construct ML models, as summarized in Table S1 in SI.

### 3.4. Reasonability of the Data Partitioning after Modeling

**Figure 5:**
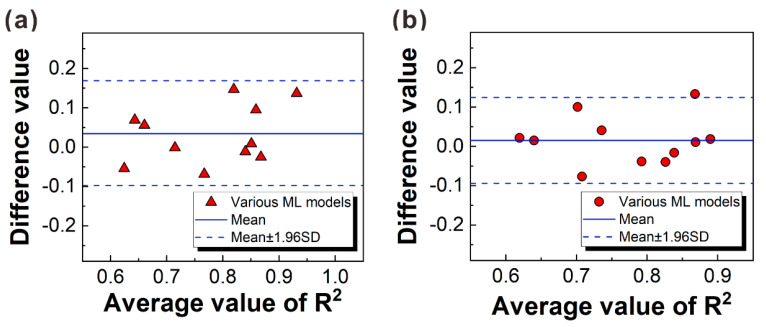
Bland-Altman plot. (**a**) The *R*^2^ values of the training set and test set after normalization and/or Box-Cox transformation; (**b**) the *R*^2^ values after transformation and antitransformation on the test dataset. Mean represents the average value of the difference (Mean_a_ = 0.0346, Mean_b_ = 0.0152), and SD represents the standard deviation of the difference (SD_a_ = 0.0684, SD_b_ = 0.0556).

Figure 5 presents the Bland-Altman plot using the *R*^2^ value as an indicator, and the impact of data partitioning on the accuracy of different models was further investigated. The results indicated that these models established using the partitioned dataset were reasonable and there was no overfitting or underfitting phenomena. It also proved that the inverse transformation of the dataset did not affect the accuracy of the models. More details are presented in SI. The evaluation indicator was calculated by

(1) $$R^{2}=1-\frac{{\sum_{i=1}^{m}\left(y_{i}^{\prime }{-y}_{i}\right)}^{2}}{{\sum_{i=1}^{m}\left(\bar{y_{i}}{-y}_{i}\right)}^{2}}$$

where the *R*^2^ value is generally used to evaluate the conformity between the predicted value and the actual value, and its value ranges from 0 to 1. The larger the *R*^2^ value, the better the model fitting effect.

## 4. Finite Element Analysis of Effects of Surface Quality and Pore Features

**Figure 6:**
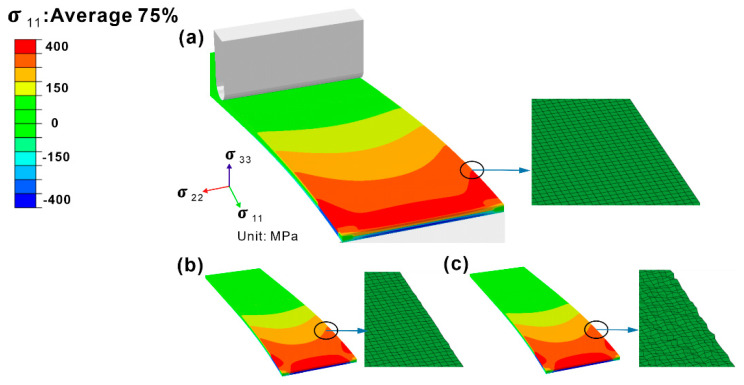
The normal stress distribution of as-built specimens with (**a**) smooth surfaces, (**b**) rough surfaces (Ra = 0.2 µm), and (**c**) rough surfaces (Ra = 0.4 µm), simulated by FEA (deflection = 0.65 mm).

It has been proven that the fracture of cantilever specimens is attributed to normal stress rather than shear stress in bending conditions [61]. The simulation results also confirmed that the shear stress was much smaller than the normal stress. Thus, the normal stress ($\sigma_{11}$) defined in Figure 6 was adopted for the FEA presented below.

### 4.1. The Influence of Surface Quality

Although the specimen underwent machining and polishing treatments to obtain a relatively smooth surface, one may argue that there may be an influence of surface quality—similar or even stronger correlations between fatigue life and surface quality. The potential influence of the surface roughness (Ra) on the maximum normal stress ($\sigma_{11}^{max}$) defined by the maximum value of the normal stresses in the cantilever beam specimens under a deflection of 0.65 mm was estimated using FEA. Figure 6 presents σ_11_ distributions in the FEA-simulated specimens with different Ra values (corresponding to the fluctuations of the grid nodes) experimentally determined.

**Figure 7:**
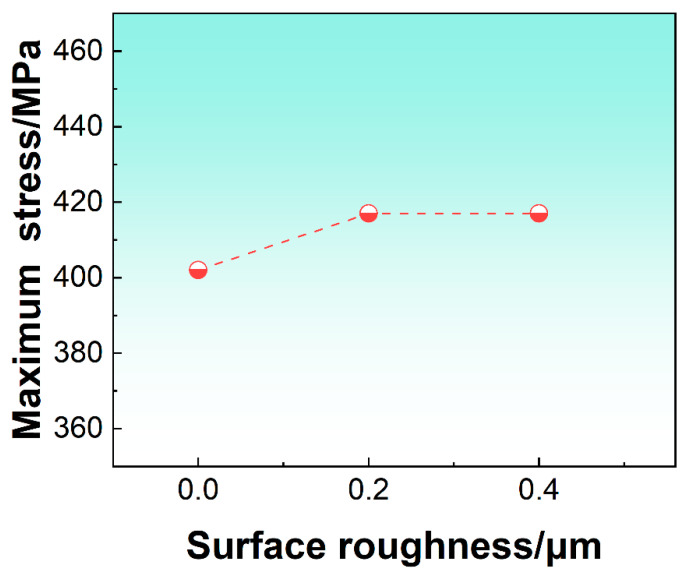
The comparison results of the maximum normal stress distribution in the fixed end of the specimen with different surface roughness values.

Figure 7 summarizes a comparison of the $\sigma_{11}^{max}$ values in the fixed end of the specimen with different Ra under a deflection of 0.65 mm estimated by FEA. During the bending process, three regions with different stress states appeared in the cross section of the cantilever beam specimen, including the tensile stress region, the neutral plane region, and the compressive stress region. The $\sigma_{11}^{max}$ values were calculated as 402 MPa for the smooth surfaces, 417 MPa for the rough surfaces (Ra = 0.2 µm), and 417 MPa for the rough surfaces (Ra = 0.4 µm), respectively. The results showed that when the Ra value was reduced down to the range from 0.2 μm and 0.4 μm after mechanical polishing and electropolishing, the $\sigma_{11}^{max}$ value at the fixed end along the beam direction on the cross section did not change significantly, as compared with the specimen with a smooth surface. Therefore, the surface roughness of the beam specimen ranging from 0.2 μm to 0.4 μm through mechanical polishing and electropolishing effectively reduced the suspected influence of surface quality on the fatigue life of the beam specimen under bending load. The influence of surface roughness as a variable on fatigue life can be ruled out in this study.

### 4.2. Analysis of the Interaction of Pore Feature and Stress Field under Bending Load

**Figure 8:**
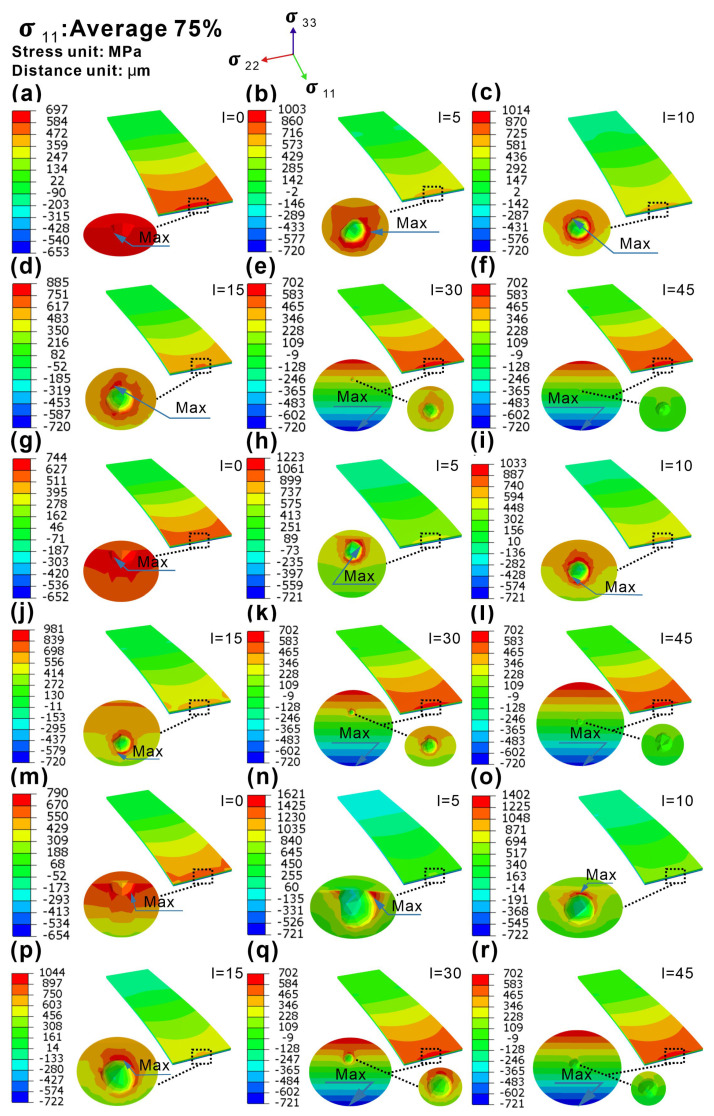
The normal stress distribution of as-built specimens with different features of pores along cantilever specimens was simulated through FEA (deflection = 0.65 mm). Specimens with (**a**–**f**) 4 µm pore diameter, (**g**–**l**) 8 µm pore diameter, and (**m**–**r**) 16 µm pore diameter. l = x represents the distance (x) from the center of the pore to the surface along the thickness direction, such that l = 15 μm means the pore is located at the place with a distance to the surface at the fixed end of the specimen along the thickness direction. All the circular insets in the figures indicate the maximum normal stress on the cross section of the specimen with the pore.

Our previous research [20] indicated that the bending fatigue life of the LPBF-fabricated Inconel 718 alloy was closely related to the pores. For the bending fatigue test of a cantilever beam, the distribution of stress across the entire cross section is uneven in addition to that along the beam direction. Thus, it is necessary to consider the interaction between pore features and stress fields. After excluding the influence factor of surface quality in Section 4.1, here, we further quantitatively analyze the influence of pore features on the stress distribution across the thickness. The FEM model of spherical defects with three typically different diameters and six different locations interacting was established, and the σ_11_ distribution of each beam was calculated and is shown in Figure 8. The circular inset in each figure shows the $\sigma_{11}^{max}$ value at the cross section in the fixed end. The existence of pores caused the $\sigma_{11}^{max}$ at the fixed end to change with the location and size of the pores. According to the $\;\sigma_{11}^{max}$ values in the beam calculated by the FEA in Figure 8a–d,g–j,m–p, it can be found that the pores weakened the uniformity of the specimen, and the stress concentration occurred near pores on the surface or subsurface. It is worth noting that when a pore exists in the specimen matrix, its $\sigma_{11}^{max}$ occurs on the specimen surface, and $\sigma_{11}$ near the pore is significantly smaller than that $\sigma_{11}^{max}$ on the specimen surface, as shown in Figure 8e,f,k,l,q,r.

**Figure 9:**
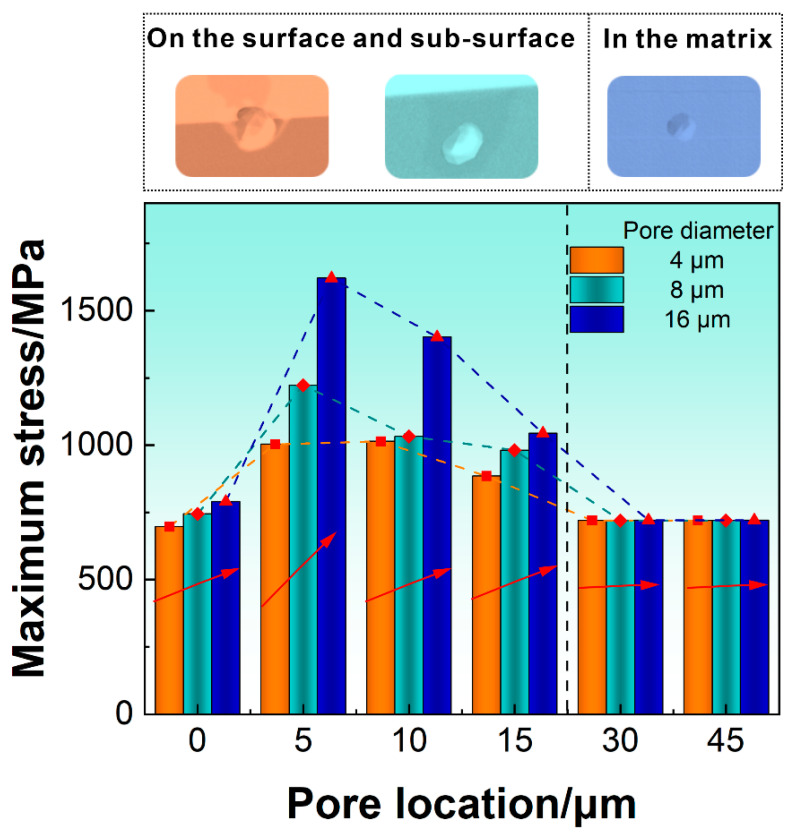
Variation of the maximum stress in the beam specimens containing pores with locations (distance to the specimen surface) and sizes of the pores.

Based on the $\sigma_{11}^{max}$ values of the as-built specimens with different pore features in Figure 8, we established the relationship between the $\sigma_{11}^{max}$ value and the pore feature of the present LPBF-fabricated Inconel 718 specimens, as shown in Figure 9. The analysis results of the interaction between pore features and stress fields are summarized below.

1. For the specimen with pores on the surface (0 μm) and subsurface (5, 10, and 15 μm), $\sigma_{11}^{max}$ increased with the increasing pore diameter from 4 μm to 16 μm. Among them, for the specimen with pores on the subsurface (5 μm), the increasing degree of $\sigma_{11}^{max}$ became relatively large with the increase in pore diameter. However, for the specimen with pores in the matrix (30 μm and 45 μm), $\sigma_{11}^{max}$ hardly changed with the increase in pore diameter, as shown by the red arrows in Figure 9.
2. For specimens with three different pore diameters (4, 8, and 16 μm), $\sigma_{11}^{max}$ significantly increased as the pore location increased from 0 μm (on the surface) to 5 μm (on the subsurface).
3. For specimens with pore diameters of 4, 8, and 16 μm, as the pore location increased from 5 μm to 10 μm, $\sigma_{11}^{max}$ slightly increased for specimens with a pore diameter of 4 μm. $\sigma_{11}^{max}$ slightly decreased for specimens with pore diameters of 8 μm and 16 μm, and the reduction in $\sigma_{11}^{max}$ for specimens with a pore diameter of 16 μm was the largest.
4. $\sigma_{11}^{max}$ of all specimens with pore diameters of 4, 8, and 16 μm showed a decreasing trend as the pore location increased from 10 μm to 15 μm. The reduction in $\sigma_{11}^{max}$ of the 16 μm pore diameter specimen was also the largest.
5. As the distance continued to increase toward the neutral plane along the thickness direction (30 μm), $\sigma_{11}^{max}$ occurred on the specimen surface and significantly decreased, which was similar to that in the specimen with pores on the specimen surface. When the pore location became 45 μm, $\sigma_{11}^{max}$ almost remained at a constant value at a location of 30 μm and did not change much.

Based on the above analysis results of the interaction between pore features and stress fields and combined with previous research [20], it is suggested that the bending fatigue properties of the LPBF-fabricated Inconel 718 alloy are closely related to the location, size, and quantity of the pores within a certain critical location threshold. The pores lead to a significant scattering in the bending fatigue properties of the LPBF-fabricated Inconel 718 alloy. The increase in the size and the number of pores and the decrease in the distance from the center of the pore to the surface of specimens can promote fatigue crack initiation and thus decrease the fatigue life.

## 5. Prediction Results and Discussion

### 5.1. Prediction Results of Fatigue Life

The small dataset of pore-influenced fatigue life initially used to train the ML models was composed of twenty-six data points. Note that the defect information of pores was mainly considered in the study.

**Figure 10:**
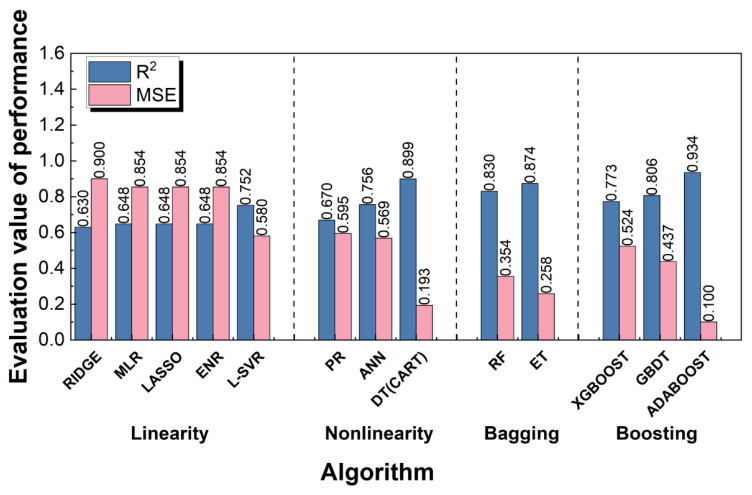
Evaluation performance of each ML algorithm on the test set.

Figure 10 reports the performance of each ML algorithm in predicting pore-affected fatigue life, where the *R*^2^ evaluation indicator (Equation (1)) was adopted. Here, the mean square error (*mse*), being sensitive to outliers, was also used to evaluate the prediction accuracy of models, and the *mse* value was calculated by

(2) $$mse=\frac{1}{m}{\sum_{i=1}^{m}\left(y_{i}^{\prime }{-y}_{i}\right)}^{2}$$

where $y_{i}$ is the actual value, $y_{i}^{\prime }$ is the predicted value, and *m* is the number of samples in the dataset. The range of the *mse* evaluation index is [0, +∞). The *mse* values were normalized to the range of [0.1, 0.9] to prevent the occurrence of extreme values and facilitate a better representation on the coordinate axis, represented by *MSE*.

An overview of the evaluation results in Figure 10 shows that the algorithms for ensemble learning categories were generally superior to the algorithms for linear and nonlinear regression categories. This may have been caused by the enhancement of the weak learners, in which the ADABOOST algorithm had the best evaluation effect (*R*^2^ = 0.934, *MSE* = 0.100), and the RIDGE algorithm had the worst evaluation effect (*R*^2^ = 0.630, *MSE* = 0.900). Detailed information on each category of algorithms is provided below.

1. Category of linear regression: For this category, the evaluation results of the model shown in Figure 10 are further discussed and analyzed. The L-SVR algorithm had the highest fitting accuracy (*R*^2^ = 0.752, *MSE* = 0.580) on the test set, while the fitting accuracy of the MLR model (*R*^2^ = 0.648, *MSE* = 0.854) on the test set was not ideal, and this study only reached the qualified level (*R*^2^ = 0.6). Since multicollinearity issues generally influence the predictive ability of models in linear regression, three regularization algorithms were proposed to check and address multicollinearity issues, including the LASSO algorithm (*L*1 regularization), the RIDGE algorithm (*L*2 regularization), and the ENR algorithm (*L*1 and *L*2 regularizations). The results in Figure 10 show that the accuracy of the three regularization models was not significantly improved compared to the MLR model. Among them, the fitting accuracy of the RIDGE algorithm decreased, which may have resulted from over-regularization. Thus, it is believed that there may not have been a multicollinearity problem in the small dataset, and the three regularization methods could not significantly improve the performance of the MLR model. In addition, the use of SVR was also investigated. However, when polynomial kernel functions, Gaussian kernel functions, or S-type kernel functions for model training were used, the evaluation results of the SVR-built model did not reach the qualified level. Ultimately, the L-SVR algorithm was selected for the model establishment, achieving a good fitting accuracy and being the best algorithm in the linear category. Zhao et al. [62] showed that the SVR algorithm had better generalization ability than the MLR algorithm in predicting the toxic activity of different datasets. Luo et al. [20] also demonstrated that the SVR algorithm was more suitable for fatigue life prediction of a small dataset than the MLR and RIDGE algorithms. This is consistent with the results of the above analysis.
2. Category of nonlinear regression: Figure 10 also shows that the DT algorithm (*R*^2^ = 0.899, *MSE* = 0.193) had the highest fitting accuracy among the algorithms for this category, followed by the ANN algorithm (*R*^2^ = 0.756, *MSE* = 0.569), and finally, the PR algorithm (*R*^2^ = 0.670, *MSE* = 0.595). Although the PR algorithm was not sufficient to obtain a satisfactory prediction model, when the complete polynomial was used for fitting directly, the accuracy of the fitting improved after removing the combination term involving the feature itself. On the other hand, the ANN algorithm improved the fitting *R*^2^ value up to 0.756. The DT algorithm is rarely used for small datasets in predicting fatigue life, but research results showed that the *R*^2^ value of the DT algorithm was significantly improved to 0.899, and there was no overfitting phenomenon that often appears in this algorithm. Although the ANN algorithm is known for its strong robustness and is often used to establish prediction models, the above analysis shows that the DT algorithm is superior to the ANN algorithm in the prediction problem of pore-affected fatigue life.
3. Bagging category: In the ensemble learning algorithms of this category, each learner has a parallel relationship. First, the RF algorithm used here achieved a better fitting accuracy of *R*^2^ = 0.830. Then, the corresponding prediction model was trained using the ET algorithm with a better generalization ability. Figure 10 shows that the fitting accuracy of the ET algorithm (*R*^2^ = 0.874, *MSE* = 0.258) was better than that achieved by the RF algorithm (*R*^2^ = 0.830, *MSE* = 0.354).
4. Boosting category: Initially, the CART algorithm was used as the base learner, which was later enhanced by using the GBDT algorithm to build the predictive model, resulting in a good fitting effect (*R*^2^ = 0.806, *MSE* = 0.437). Subsequently, the XGBOOST algorithm was utilized to build the ML model, which had a lower overfitting probability. The evaluation result of the test set for this model was not significantly different from that (*R*^2^ = 0.773, *MSE* = 0.524) of the GBDT algorithm. Finally, the ADABOOST algorithm was adopted, which produced the best fitting (*R*^2^ = 0.934, *MSE* = 0.100) among all the algorithms.

Based on the above analysis results, it can be found that the *R*^2^ value obtained by the ADABOOST algorithm outperformed that obtained by other ML algorithms when working with a small dataset, although other algorithms were also effective. It is worth noting that even with limited quantities of data, data-driven analytical models can still achieve reasonably high accuracy, which suggests that applying ML techniques to small datasets for predicting fatigue life is theoretically reasonable.

### 5.2. Visualization of Prediction Results

**Figure 11:**
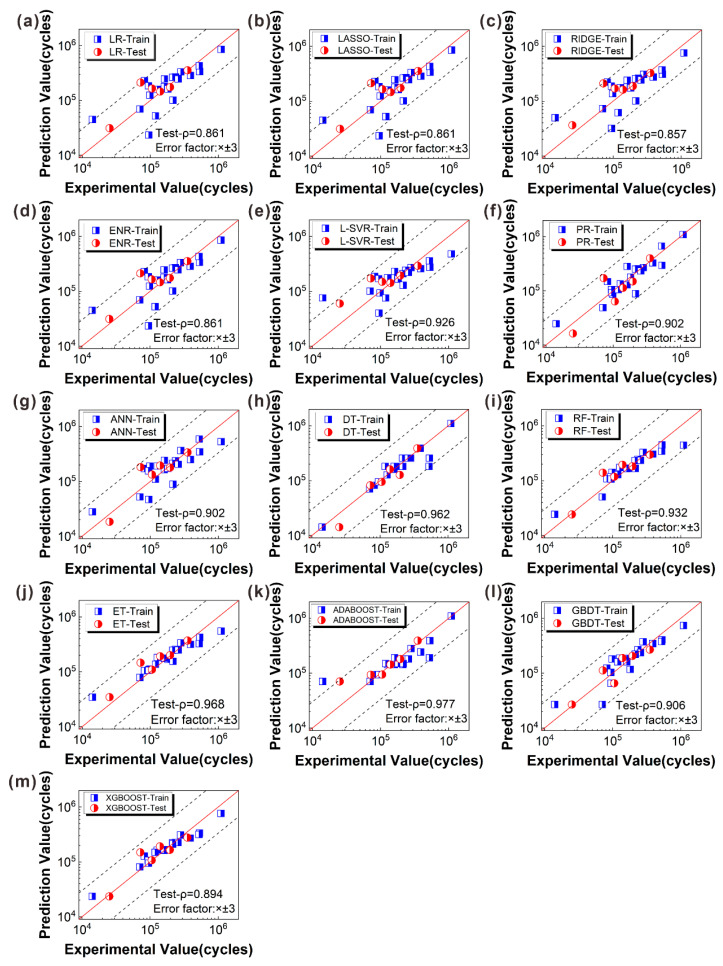
The comparison between the experimental and predicted values. (**a**–**e**) Linear category, (**f**–**h**) nonlinear category, (**i**,**j**) bagging category, (**k**–**m**) boosting category.

Figure 11 displays the comparison between the experimental fatigue life for miniature specimens of AM-fabricated Inconel 718 and the predicted fatigue life using ML algorithms. The results indicate that only a few data points fell outside of the margin of the error band, with most other data points falling within the threefold error band. However, it should be noted that the number of data points was limited and tended to deviate from the error band when the fatigue life was less than 1 × 10^5^ cycles. This suggests that some prediction models established by the corresponding algorithms were not ideal for predicting fatigue life within this interval, except for the DT, RF GBDT, and XGBOOST algorithms.

When the fatigue life was more than 1 × 10^5^ cycles, the ADABOOST algorithm generated the best evaluation effect on the test set, thereby providing a useful reference for future algorithm selection. It is worth noting that the dataset displayed a considerable scatter in terms of fatigue life. He et al. [18] showed that the probability distribution of fatigue life resulted in a significant dispersion of experimental data. In particular, even specimens with comparable surface roughness and stress amplitude may exhibit different fatigue lives, which is an inherent feature of fatigue testing.

In addition to providing an intuitive expression, the Pearson correlation coefficient (*ρ*) was utilized to quantitatively assess the degree of linear correlation between the experimental and predicted values on the test set. Figure 11 illustrates that the *ρ* value obtained through Equation (3) exceeded 0.85 for each algorithm. This indicates a strong linear correlation between the experimental and predicted values on the test set, highlighting a favorable prediction effect. The *ρ* value is given by

(3) $$\rho =\frac{cov\left(y,Y\right)}{\sigma_{x}\sigma_{Y}}$$

where *cov*(*y, Y*) is the covariance of the experimental and predicted values, *σ_y_* is the standard deviation of the predicted value, and *σ_Y_* is the standard deviation of the experimental value.

### 5.3. Verification of Prediction Results

**Figure 12:**
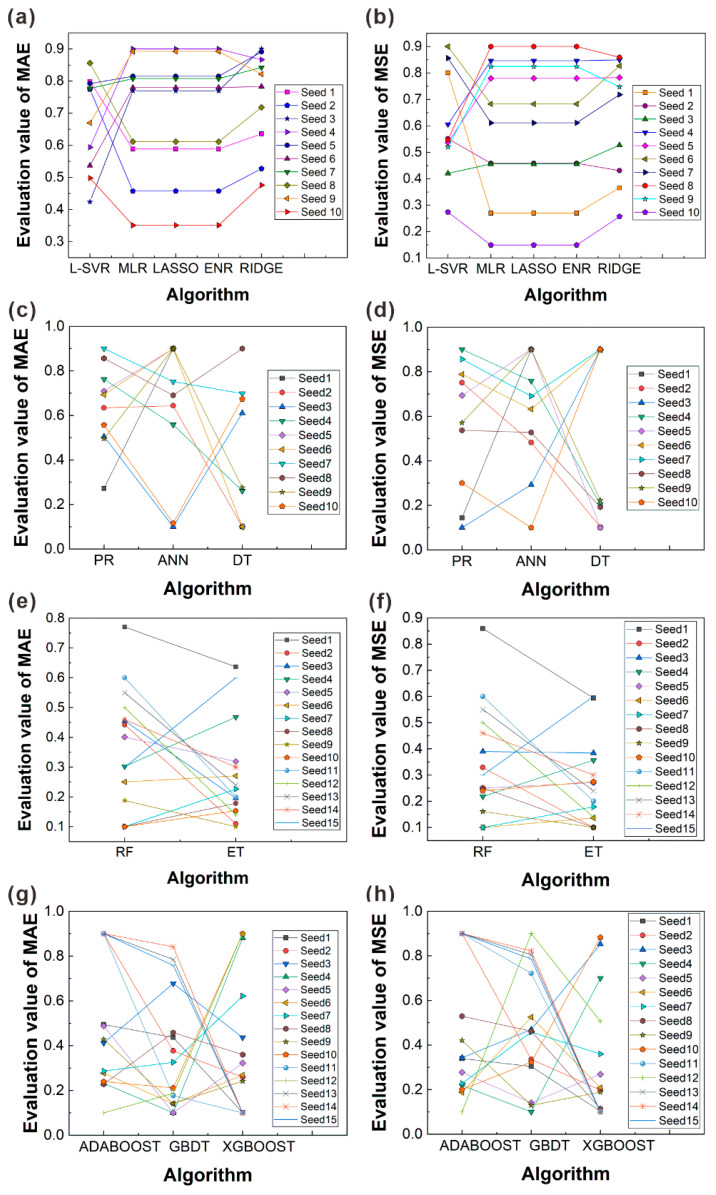
Performance verification of each algorithm under different random seeds and evaluation indicators. (**a**,**b**) Linear regression category; (**c**,**d**) nonlinear regression category; (**e**,**f**) bagging algorithm category; (**g**,**h**) boosted algorithm category. Here, the random seeds of (**a**–**d**) are 5, 15, 25, 35, 45, 55, 65, 75, 85, and 95, and those of (**e**–**h**) are 5, 15, 25, 35, 45, 55, 65, 75, 85, 95,100, 500, 1000, 1500, and 2000.

Figure 12 presents the performance verification of each algorithm under different random seeds and evaluation indicators. Based on the above evaluation outcomes in Section 4.1, one can find that when the algorithm evaluations suffer from accidental testing problems, the evaluation results are not convincing. As a result, it is still insufficient to sort the various algorithms. The following discusses the validation ideas.

The previous evaluation results were verified and corrected using the principle of “high probability”. Specifically, this work created various virtual test sets by changing different random seeds (each seed represented different partitioning results) multiple times to evaluate the algorithm performance on the entire small dataset. The final ranking of the algorithms was then determined based on the frequency of each algorithm appearing at different sort locations, resulting in more reliable evaluation outcomes. As another evaluation indicator of the algorithm, the average absolute error (*mae*) is more robust than the *mse* in handling outliers, although the update gradient remains unchanged. The *mae* value is estimated by

(4) $$mae=\frac{1}{m}\sum_{i=1}^{m}\left|y_{i}^{\prime }{-y}_{i}\right|$$

where $y_{i}$ is the actual value, $y_{i}^{\prime }$ is the predicted value, and *m* is the number of samples in the dataset. The range of the *mae* evaluation index is [0, +∞). To prevent the occurrence of extreme values and facilitate a better representation on the coordinate axis, the *mae* values were also normalized to the range of [0.1, 0.9], represented by *MAE*.

Figure 12a,b present the performance verification of the linear-category algorithms under ten random seeds. The results indicate that for each random seed, the evaluation values of the MLR, LASSO, and ENR algorithms are connected by a horizontal straight line, suggesting a similar accuracy among the three algorithms. Furthermore, by analyzing the trend of connecting lines between L-SVR and MLR evaluation values, about 55% of the lines exhibit a downward trend. The result provides additional evidence for the better performance of L-SVR over MLR, consistent with previous evaluations. Finally, by observing the trend of connecting lines between ENR and RIDGE evaluation values, about 75% of the lines display an upward trend, further confirming the ranking of algorithms for the linear category (L-SVR > MLR ≈ LASSO ≈ ENR > RIDGE).

Figure 12c,d present the performance verification of the nonlinear-category algorithms under ten random seeds. A statistical analysis of connecting lines between PR and ANN evaluation values was conducted. It was found that about 60% of the lines exhibited a downward trend, indicating that the ANN algorithm performed better than the PR algorithm. A comparison of connecting lines between ANN and DT evaluation values revealed that about 56% of the lines showed a downward trend, further demonstrating that the ranking of algorithms for the nonlinear category was DT > ANN > PR.

Figure 12e,f show the performance verification of the ensemble learning algorithms for the bagging category. As the difference in fitting accuracy between the two algorithms was minor, to obtain more reliable verification results, on top of ten random seeds, five random seeds were added to obtain different virtual test sets. By comparing the trend of connecting lines between the RF and ET evaluation values, it was found that about 63% of the lines showed a downward trend, indicating that the ET algorithm outperformed the RF algorithm.

Figure 12g,h show the performance verification of the ensemble learning algorithms for the boosting category under fifteen random seeds selected randomly. The statistical observation of connecting lines between the GBDT and XGBOOST evaluation values revealed that about 60% of the lines displayed a downward trend, revealing that the XGBOOST algorithm was superior to the GBDT algorithm. However, the result was inconsistent with the results obtained from the test set in Figure 12, where the evaluation results of the two algorithms were similar (*R*^2^_XGBOOST_ = 0.773, *MSE*_XGBOOST_ = 0.524, *R*^2^_GBDT_ = 0.806, *MSE*_GBDT_ = 0.437), and correct conclusions cannot be obtained from a visual comparison. The verification demonstrated that the generalization ability of the GBDT algorithm was lower than that of the XGBOOST algorithm with the small dataset. Next, a comparison of connecting lines between the ADABOOST and GBDT evaluation values indicated that about 63% of the lines showed an upward trend. Furthermore, the analysis of the relationship between the ADABOOST and XGBOOST algorithms revealed that 60% of the connecting lines also exhibited an upward trend. Thus, the updated rankings for these algorithms were ADABOOST > XGBOOST > GBDT.

### 5.4. Ranking Results of Thirteen Algorithms

**Figure 13:**
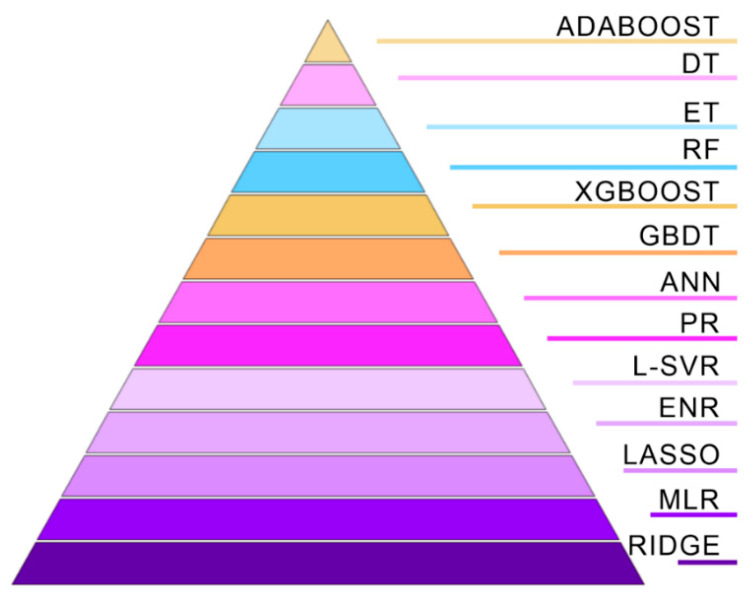
The ranking results of different algorithms.

Based on the discussion results in Section 5.3, Figure 13 summarizes the ranking results of the different algorithms utilized to predict the pore-affected fatigue life of AM-fabricated Inconel 718. To demonstrate the evaluation results in Section 4.1 in conjunction with Figure 13, Figure S3 in SI also summarizes the evaluation values of different algorithms under different indicators.

The result shows that all algorithms can effectively predict the pore-affected fatigue life of AM-fabricated components, resolving the challenge of fatigue life prediction in a small dataset. Meanwhile, this approach also assists in evaluating the impact of pores on fatigue life and guides the selection of ML algorithms for such applications. However, it remains uncertain whether the ability of the model to predict a broader range of datasets is feasible. This uncertainty can be attributed to the smaller number of data points trained. Thus, future research should conduct more fatigue tests at varying strain amplitudes using miniature specimens from LPBF-fabricated components to obtain more accurate results and better understand the predictive capabilities of the models for wider ranges of data [32].

Moreover, it is essential to continue developing appropriate research methods combining traditional experimental techniques with intelligent data analysis techniques to improve experimental efficiency and reduce error rates [63]. Such efforts will enhance the field of predictive analytics, provide more reliable and accurate predictions for complex materials, and accelerate the development of AM technologies.

## 6. Conclusions

1. Among the thirteen popular ML algorithms investigated, all had *R*^2^ values above 0.6. Compared to previous studies on the influence of pores on fatigue life on small datasets, the predictive performance of the algorithms was improved. The ADABOOST algorithm from the boosting category exhibited the best fitting accuracy (*R*^2^ = 0.934, *MSE* = 0.100) for fatigue life prediction of the AM-fabricated Inconel 718 using the small dataset, followed by the DT algorithm (*R*^2^ = 0.899, *MSE* = 0.193) in the nonlinear category.
2. The DT, RF, GBDT, and XGBOOST algorithms can well predict the fatigue life of the AM-fabricated Inconel 718 within the range of 1 × 10^5^ cycles compared to others investigated in this study.
3. By subjecting the prediction models to twenty or thirty verifications using *R*^2^ and *MSE* evaluation indicators, the ranking results of various ML algorithms for fatigue life prediction on a small dataset were obtained as follows: ADABOOST > DT > ET > RF > XGBOOST > GBDT > ANN > PR > L-SVR > ENR ≈ LASSO ≈ MLR > RIDGE. The fluctuation trends of the straight lines representing the performance of each algorithm informed these rankings, providing more reliable and robust conclusions.

## Figures and Tables

**Table 1:** Input and output ranges of various features in the dataset [20,27].

	Input and Output Values	Max	Min	Mean	Std
Inputs	Strain amplitude (*ε*/%)	1.21	0.17	0.43	0.28
	Pore diameter (*d*/µm)	17.87	0.00	4.52	4.72
	Pore amount (*m*/piece)	4.00	0.00	1.43	1.20
	Pore location—all data (*l*/µm)	1.00 × 10^10^	0.00	2.14 × 10^9^	4.18 × 10^9^
	Pore location—excluding data with zero number of pores (*l*/µm)	50.00	0.00	16.19	15.38
Outputs	Fatigue life (*N*/cycles)	1.10 × 10^6^	1.42 × 10^4^	2.30 × 10^5^	2.18 × 10^5^

**Table 2:** The details of ML algorithms for fatigue life prediction.

ML Models	Mathematical Model	Other Information	References
MLR	$Y\left(x_{i}\right)=\beta_{0}+\cdots +\beta_{k}x_{k}=\beta^{T}x_{i}$	An optimal combination of multiple independent variables	[39]
LASSO	${Cost}_{LASSO}\left(\beta \right)=\sum_{i=1}^{k}{\left(y_{i}-Y\left(x_{i}\right)\right)}^{2}+\lambda {\left\|\beta \right\|}_{1}$	Penalty coefficient (*λ =* 4.77 × 10^−9^)	[40]
RIDGE	${Cost}_{RIDGE}\left(\beta \right)=\sum_{i=1}^{k}{\left(y_{i}-Y\left(x_{i}\right)\right)}^{2}+\lambda {\left\|\beta \right\|}_{2}^{2}$	Penalty coefficient (*λ* = 0.408)	[41,42]
ENR	${Cost}_{ENR}\left(\beta \right)=\sum_{i=1}^{k}{\left(y_{i}-Y\left(x_{i}\right)\right)}^{2}\;+\lambda \rho {{\left\|\beta \right\|}_{1}+\frac{\lambda \left(1-\rho \right)}{2}\left\|\beta \right\|}_{2}^{2}$	Penalty coefficient (*λ* = 1.0 × 10^−11^) Hybrid parameter (*ρ =* 0.5)	[43]
L-SVR	$f\left(x\right)=wx+b=\sum_{i=1}^{k}\left(\alpha_{i}^{1}-\alpha_{i}^{2}\right)x_{i}^{T}x+b\;f\left(x\right)=w\psi \left(x\right)+b\;=\sum_{i=1}^{k}\left(\alpha_{i}^{1}-\alpha_{i}^{2}\right)k\left(x,x_{i}\right)+b$	Linear kernel function	[44]
DT	$Gini\left(D\right)=1-\sum_{k=1}^{\left|y\right|}p_{k}^{2}$	The classification and regression tree (CART) was employed	[45,46]
ANN	$f\left(x\right)=\frac{1}{1+e^{-x}}$	The sigmoid activation function	[24,47]
PR	$F\left(x\right)=ax_{1}^{2}+bx_{2}^{2}+\cdots +cx_{1}x_{2}+dx_{2}x_{i}\;\;+\cdots +ex_{1}+fx_{2}+\cdots +g$	The curve can better capture the nonlinearity	[48]
ET	—	A selection of features in a more varied and diverse manner than the RF algorithm	[49]
RF	—	The algorithm is relatively more intricate than other ML algorithms	[50,51]
ADABOOST	$\alpha_{m}=\frac{1}{2}ln\left(\frac{1-e_{m}}{e_{m}}\right)\;F\left(x\right)=\sum_{m=1}^{M}\alpha_{m}G_{m}\left(x\right)$	It is widely considered one of the best learning algorithms	[52,53]
XGBOOST	$\phi \left(x_{i}\right)=\sum_{i=1}^{K}f_{k}^{*}\left(x_{i}\right),f_{k}^{*}\in F$	The algorithm adds a regular term to the loss function to control the complexity of the model	[54]
GBDT	$f_{M}\left(x\right)=\sum_{m=1}^{M}T\left(x;\theta_{m}\right)$	The least squares method was employed in this study to measure the regression effect of the regression tree	[55,56,57]

## Data Availability

The data presented in this study are available on request from the corresponding author.

## Supplementary Materials

The following supporting information can be downloaded at: https://www.mdpi.com/article/10.3390/ma16196606/s1, Figure S1: Process of finding the optimal solution. (a) The search process without normalization, and (b) the search process with normalization; Figure S2: Gaussian distribution histograms and kernel density curves of the (a) strain amplitude (%), (b) pore diameter (µm), (c) pore amount (piece), (d) pore location (µm), and (e) fatigue life (cycles). The Q-Q plots of the (f) strain amplitude (%), (g) pore diameter (µm), (h) pore amount (piece), (i) pore location (µm), and (j) fatigue life (cycles); Figure S3: The evaluation values of different algorithms under different indicators. Table S1: Definition of various ML algorithm categories and summary of the pre-processed data used.
