# Supplementary material for: A Comparative Investigation of Machine Learning Algorithms for Pore-Influenced Fatigue Life Prediction of Additively Manufactured Inconel 718 Based on a Small Dataset
Source: Materials (Basel). 2023 Oct 9;16(19):6606. doi: 10.3390/ma16196606 (PMC10574268; doi:10.3390/ma16196606)
Supplement: Supplementary file 1 [file materials-16-06606-s001.zip › materials-2602902-supplementary.pdf]

# Supplementary Information

## A comparative investigation of machine learning algorithms for pore-influenced fatigue life prediction of additively-manufactured Inconel 718 based on a small dataset

B.L. Hu[1,2], Y. W. Luo[3], B. Zhang[3], G.P. Zhang[1,*]

[1] Shenyang National Laboratory for Materials Science, Institute of Metal Research, Chinese Academy of Sciences, 72 Wenhua Road, Shenyang 110016, China

[2] School of Materials Science and Engineering, University of Science and Technology of China, Shenyang 110016, China

[3] Key Laboratory for Anisotropy and Texture of Materials, Ministry of Education, School of Materials Science and Engineering, Northeastern University, 3-11 Wenhua Road, Shenyang 110819, P. R. China

*Corresponding author. *E-mail address:* gpzhang@imr.ac.cn

### 1. The simple max-min normalization method

The statistical values of the K-S value (D-value) and the P-value under hypothesis testing were obtained from Figure 3, and both were used to describe differences in the distribution of the two datasets. The D and P values obtained are as follows: D-value ($D_{\text{strain amplitude}} = 0.167$, $D_{\text{pore diameter}} = 0.167$, $D_{\text{pore amount}} = 0.183$, $D_{\text{pore location}} = 0.200$, $D_{\text{fatigue life}} = 0.283$) and P-value ($P_{\text{strain amplitude}} = 0.997$, $P_{\text{pore diameter}} = 0.997$, $P_{\text{pore amount}} = 0.989$, $P_{\text{pore location}} = 0.972$, $P_{\text{fatigue life}} = 0.781$). If the D-value is small and the P-value is large, the null hypothesis of the K-S test can be accepted, that is, the distribution of the two datasets is consistent. The results indicate that the distribution of the training and test sets is approximately consistent, thereby validating the reasonableness of the dataset division.

Figure S1(a) presents the objective function becoming "flat" due to differences in dimension among different types of eigenvalues. This not only increases the training time of the model but also adversely affects its predictive accuracy. To eliminate the dimensional influence among features,

* Corresponding author: gpzhang@imr.ac.cn

prevent gradient explosion, and improve the predictive performance of the ML model, the dataset needs to be normalized. It maps the dataset to the interval of [0, 1]. The optimized search process is depicted in Figure S1(b), and the max-min normalization method is shown by

$$x^* = \frac{x - min}{max - min} \tag{1}$$

where $x$ is the eigenvalue of the original dataset, and $x*$ is the eigenvalue of the normalized dataset. max and min are the maximum and minimum eigenvalues of the original dataset, respectively.

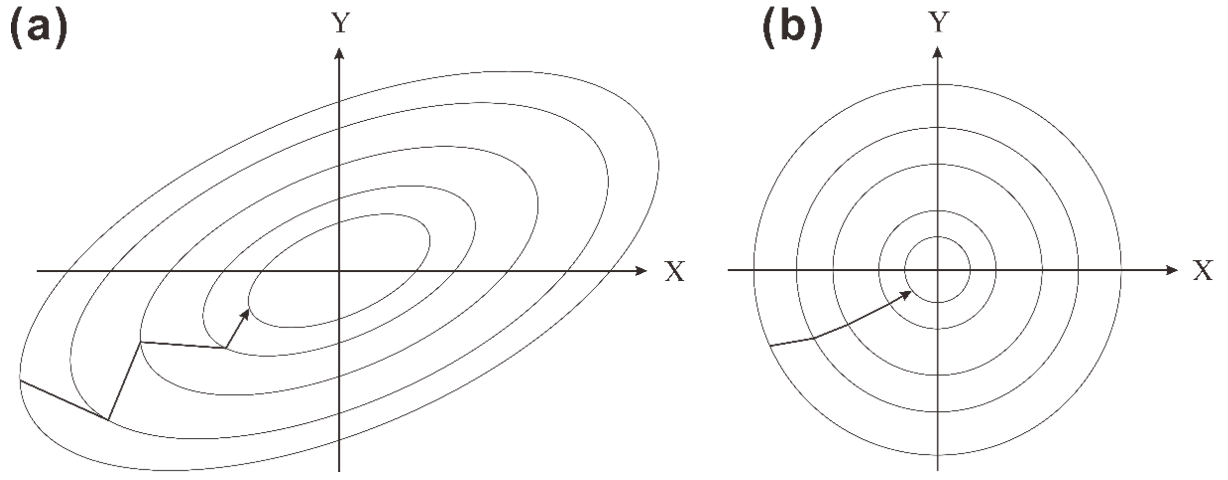


**Figure S1.** Process of finding the optimal solution. (a) The search process without normalization, and (b) the search process with normalization.

## 2. Normal transformation

The Gaussian distribution histogram and kernel density estimation (KDE) methods adopted in Figures S2(a)-(e) reveal a notable skewness issue with each normalized feature. The features of fatigue life, pore location, and strain amplitude exhibit significant skewness issues. Quantile-quantile (Q-Q) shown in Figures S2(f)-(j) evaluates the conformity between the actual and theoretical distribution of the features, and indicates that apart from pore diameter and fatigue life features, the data points of other features significantly deviate from the straight line, resulting in a non-Gaussian distribution of the normalized dataset. To make each feature more closely resemble the Gaussian

distribution, the BOX-COX transformation was conducted to correct the skewness and kurtosis problems of the dataset to make the normalized dataset more compatible with the Gaussian distribution, and the formula used is described as:

$$Y^{(\lambda)} = \begin{cases} \frac{Y^{\lambda}-1}{\lambda}, \lambda \neq 0 \\ lnY, \lambda = 0 \end{cases} \tag{2}$$

where $\lambda$ ($\lambda_{\text{strain amplitude}}$ = 0.367, $\lambda_{\text{pore diameter}}$ = 0.563, $\lambda_{\text{pore amount}}$ = 0.803, $\lambda_{\text{pore location}}$ = -0.318, $\lambda_{\text{fatigue life}}$ = 0.389) is an undetermined transformation parameter after the normalization of different features, and $\lambda$ ($\lambda_{\text{strain amplitude}}$ = -1.093, $\lambda_{\text{pore diameter}}$ = 0.563, $\lambda_{\text{pore amount}}$ = 0.803, $\lambda_{\text{pore location}}$ = 5.235, $\lambda_{\text{fatigue life}}$ = 0.141) is an undetermined transformation parameter with original features. The maximum value of the maximum likelihood function determines the size of the parameter $\lambda$. $Y$ and $Y^{(\lambda)}$ represent vectors before and after transformation, respectively.

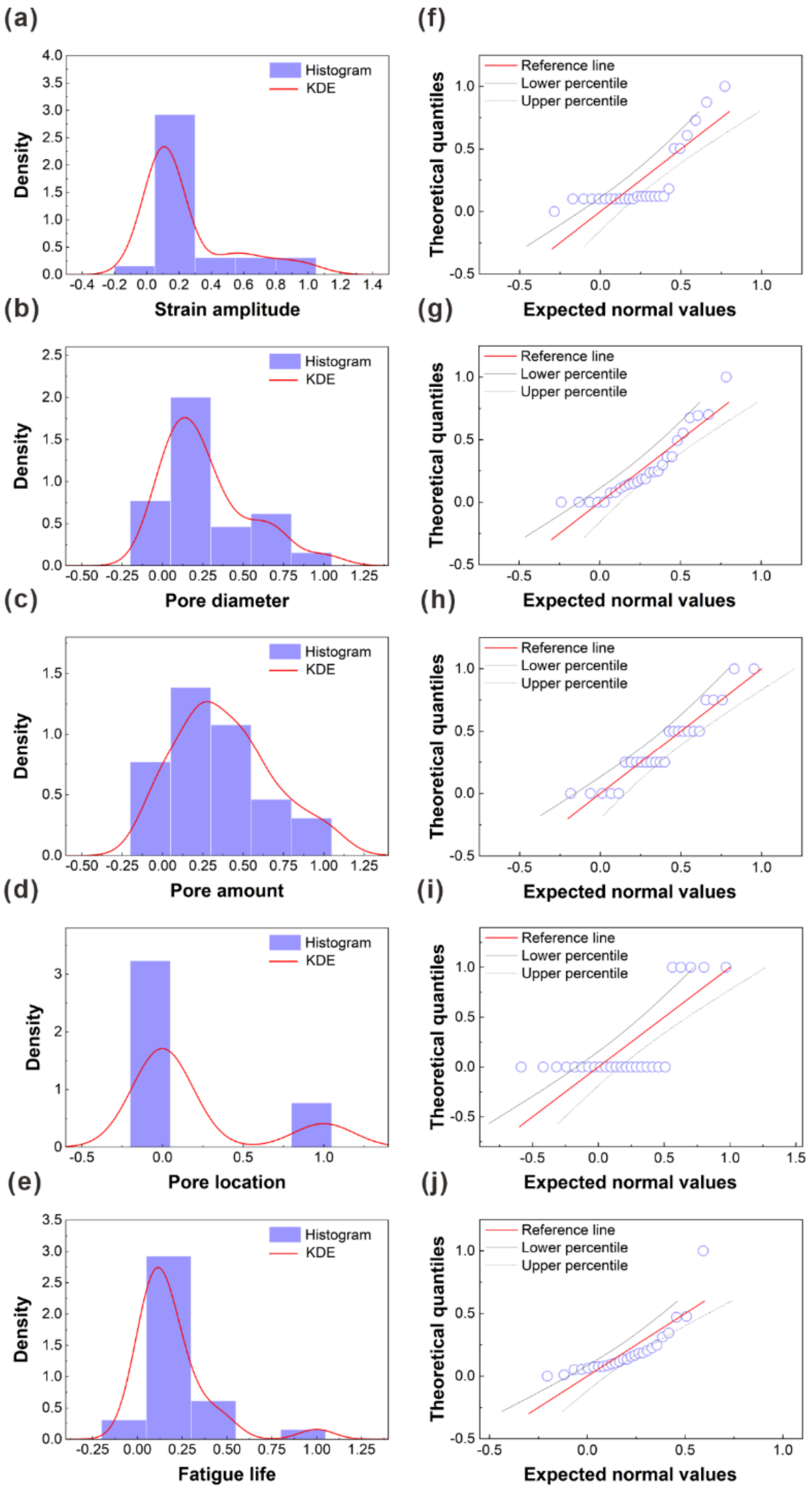


**Figure S2.** Gaussian distribution histograms and kernel density curves of the (a) strain amplitude (%), (b) pore diameter (μm), (c) pore amount (piece), (d) pore location (μm), and (e) fatigue life (cycles). The Q-Q plots of the (f) strain amplitude (%), (g) pore diameter (μm), (h) pore amount (piece), (i) pore location (μm), and (j) fatigue life (cycles).

**Table S1.** Definition of various ML algorithm categories and summary of the pre-processed data used

| Categories | Data pre-processing:<br>A: Normal transformation<br>B: Normalization and normal transformation | ML models |
|---|---|---|
| Linearity | B | MLR |
| | B | LASSO |
| | B | RIDGE |
| | B | ENR |
| | B | L-SVR |
| Nonlinearity | A | DT |
| | B | ANN |
| | B | PR |
| Bagging | A | ET |
| | A | RF |
| Boosting | B | ADABOOST |
| | A | XGBOOST |
| | B | GBDT |

## 3. Reasonability of the data partitioning after modeling

Although Figure 3 shows that the distribution of each feature in both the training and test sets is consistent before establishing the ML models, it is unclear whether this partitioning result will affect the accuracy of ML models established by different algorithms after establishing the prediction model. Figure 5(a) reveals that approximately 95% of the differences between the two types of the dataset are within the *Mean±1.96SD* range. This suggests remarkable consistency between the evaluation results of the test set and training set and also indicates that there is no overfitting or underfitting.

In Section 3.2, the dataset was normalized and Box-Cox-transformed, resulting in changes in the output fatigue life data as well. To obtain the fatigue life values of the same dimension, it is necessary to use the processing parameters of the training set to sequentially perform anti-Box-Cox

transformation and/or anti-normalization on the predicted values of fatigue life. On the test set, to evaluate the consistency between the $R^2$ value obtained after anti-normalization and/or anti-Box-Cox transformation of the fatigue life feature and those without anti-transformation, Figure 5(b) reveals that approximately 95% of the differences between the two types of the dataset are within the *Mean±1.96SD* range. It can be explained that the $R^2$ values obtained by both methods are consistent regardless of whether the inverse transformation is performed. This verifies the difference between the $R^2$ values of the prediction model after anti-transformation and those without anti-transformation.

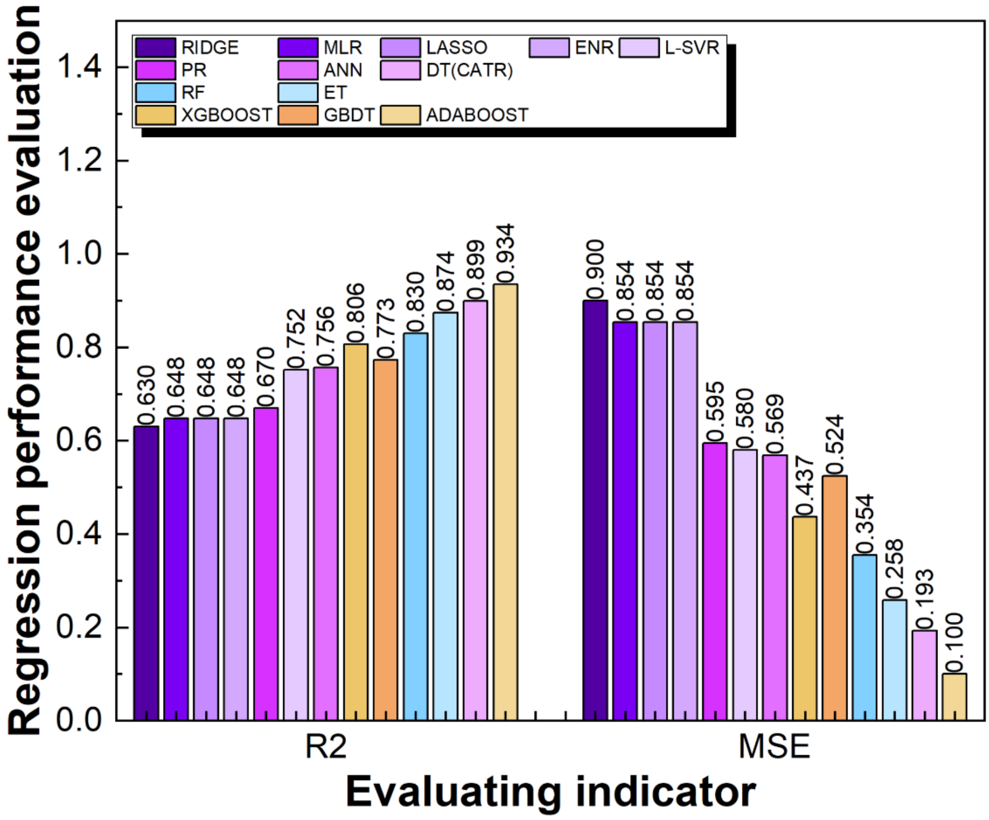


**Figure S3.** The evaluation values of different algorithms under different indicators
